# Pragmatic Strategy Empowering Paramedics to Assess Low-Risk Trauma Patients With the Canadian C-Spine Rule and Selectively Transport Them Without Immobilization: Protocol for a Stepped-Wedge Cluster Randomized Trial

**DOI:** 10.2196/16966

**Published:** 2020-06-01

**Authors:** Christian Vaillancourt, Manya Charette, Monica Taljaard, Kednapa Thavorn, Elizabeth Hall, Brent McLeod, Dean Fergusson, Jamie Brehaut, Ian Graham, Lisa Calder, Tim Ramsay, Peter Tugwell, Peter Kelly, Sheldon Cheskes, Refik Saskin, Amy Plint, Martin Osmond, Colin Macarthur, Sharon Straus, Paula Rochon, Denis Prud'homme, Simone Dahrouge, Susan Marlin, Ian G Stiell

**Affiliations:** 1 Department of Emergency Medicine Ottawa Hospital Research Institute University of Ottawa Ottawa, ON Canada; 2 Ottawa Hospital Research Institute Ottawa, ON Canada; 3 Patient Representative Owner-Hall Consulting Director-Helping Hands for India Kanata, ON Canada; 4 Paramedic Representative Hamilton Paramedic Service Hamilton, ON Canada; 5 Medical Care Analytics Canadian Medical Protective Association Ottawa, ON Canada; 6 Ottawa Paramedic Service Ottawa, ON Canada; 7 Department of Family and Community Medicine Division of Emergency Medicine University of Toronto Toronto, ON Canada; 8 Institute for Clinical Evaluative Sciences Toronto, ON Canada; 9 Children’s Hospital of Eastern Ontario Research Institute Ottawa, ON Canada; 10 Clinical Research Services, SickKids Toronto, ON Canada; 11 Knowledge Translation Program St. Michael’s Hospital Toronto, ON Canada; 12 Women’s College Research Institute Toronto, ON Canada; 13 Institut de recherche de l’Hôpital Montfort Ottawa, ON Canada; 14 Élisabeth Bruyère Research Institute Ottawa, ON Canada; 15 Clinical Trials Ontario Toronto, ON Canada

**Keywords:** cervical spine injury, Canadian C-Spine rule, immobilization, paramedic, trauma

## Abstract

**Background:**

Each year, half a million patients with a potential neck (c-spine) injury are transported to Ontario emergency departments (EDs). Less than 1.0% (1/100) of these patients have a neck bone fracture. Even less (1/200, 0.5%) have a spinal cord injury or nerve damage. Currently, paramedics transport all trauma victims (with or without an injury) by ambulance using a backboard, cervical collar, and head immobilizers. Importantly, prolonged immobilization is often unnecessary; it causes patient discomfort and pain, decreases community access to paramedics, contributes to ED crowding, and is very costly. We therefore developed the Canadian C-Spine Rule (CCR) for alert and stable trauma patients. This decision rule helps ED physicians and triage nurses to safely and selectively remove immobilization, without x-rays and missed injury. We successfully taught Ottawa paramedics to use the CCR in the field in a single-center study.

**Objective:**

This study aimed to improve patient care and health system efficiency and outcomes by allowing paramedics to assess eligible low-risk trauma patients with the CCR and selectively transport them without immobilization to the ED.

**Methods:**

We propose a pragmatic stepped-wedge cluster randomized design with health economic evaluation, designed collaboratively with knowledge users. Our 36-month study will consist of a 12-month setup and training period (year 1), followed by the stepped-wedge trial (year 2) and a 12-month period for study completion, analyses, and knowledge translation. A total of 12 Ontario paramedic services of various sizes distributed across the province will be randomly allocated to one of three sequences. Paramedic services in each sequence will cross from the control condition (usual care) to the intervention condition (CCR implementation) at intervals of 3 months until all communities have crossed to the intervention. Data will be collected on all eligible patients in each paramedic service for a total duration of 12 months. A major strength of our design is that each community will have implemented the CCR by the end of the study.

**Results:**

Interim results are expected in December 2019 and final results in 2020. If this multicenter trial is successful, we expect the Ontario Ministry of Health will recommend that paramedics evaluate all eligible patients with the CCR in the Province of Ontario.

**Conclusions:**

We conservatively estimate that in Ontario, more than 60% of all eligible trauma patients (300,000 annually) could be transported safely and comfortably, without c-spine immobilization devices. This will significantly reduce patient pain and discomfort, paramedic intervention times, and ED length of stay, thereby improving access to paramedics and ED care. This could be achieved rapidly and with lower health care costs compared with current practices (possible cost saving of Can $36 [US $25] per immobilization or Can $10,656,000 [US $7,335,231] per year).

**Trial Registration:**

ClinicalTrials.gov NCT02786966; https://clinicaltrials.gov/ct2/show/NCT02786966.

**International Registered Report Identifier (IRRID):**

DERR1-10.2196/16966

## Introduction

### Background

#### Problem

Ontario paramedic services annually transport half a million patients with a potential neck (cervical/c-spine) injury from falls or motor vehicle collisions to local emergency departments (EDs). Of these patients, 95.0% (95/100) are alert and stable and at low risk of c-spine injury. Less than 1.0% (1/100) have a c-spine fracture, and even less (1/200, 0.5%) have a spinal cord injury. Spinal cord injuries result from moderate-to-severe blunt traumas and not from minor movements occurring during transport to hospital. Regardless, current paramedic practice is to transport all such trauma victims (with or without c-spine injury) by ambulance using backboards, collars, and head immobilizers. These patients stay fully immobilized until an ED bed is made available, sometimes for as long as 3 hours. This prolonged immobilization is often unnecessary and increases patient discomfort, contributes to ED crowding, prolongs paramedic intervention times, and adds a heavy financial burden to our health care system.

#### Why C-Spine Immobilization of Low-Risk Patients May Be Unwarranted

Not only is immobilization often unnecessary, but its potential for clinical adverse effects and discomfort are also well documented [1]. Chest straps used in immobilization can have a pulmonary-restrictive effect, even in healthy nonsmokers. Immobilization on a board leads to progressively worsening pain in the head, neck, and back area, often resulting in the necessity to perform diagnostic imaging on an otherwise normal spine in the ED. The presence of a c-spine immobilization collar has been associated with hyperextension, causing spinal cord injury in patients affected by ankylosing spondylitis. In addition, c-spine collars can cause neck vein compression and increased intracranial pressure for patients with head injury, difficulty swallowing, and local skin necrosis.

We have identified three systematic reviews relevant to c-spine immobilization. A review published by Abram and Bulstrode in 2010 (comprising 32 studies) suggested there was a growing body of evidence documenting the “risks and complications of routine spinal immobilization*”* and that there was a “possibility that immobilization could be contributing to mortality and morbidity in some patient*s”* [1]. A more recent review by Sundstrom et al (220 studies) concluded that there is limited evidence supporting current c-spine immobilization practices and that large definitive randomized trials are lacking. It further concluded that the benefit of c-spine immobilization on neurological injury and spinal stability is uncertain and that there is a growing body of opinions against the use of c-spine collars [2]. The International Liaison Committee on Resuscitation (ILCOR) provides international guidelines on cardiac arrest and trauma resuscitation. In November 2015, ILCOR published a recommendation not to use routine application of c-spine collars for adults and children with blunt suspected traumatic c-spine injury (based on very low quality of evidence from 29 studies) [3].

#### Effect on Overburdened Paramedic Systems and Crowded Emergency Departments

As trauma victims need to be seen rapidly at the hospital, paramedics are given only 15 to 20 min to evaluate and treat them in the field before transport. Even for minor trauma victims, c-spine immobilization takes more than 5 min to apply, or up to 30% of the allotted field time. Unlike minor trauma victims coming to the ED by their own means of transport and commonly triaged to the waiting room area, minor trauma victims immobilized and transported by paramedics may have to wait up to 3 hours until an ED stretcher becomes available, in turn holding up the paramedic crew who then become unavailable for the next community emergency. In 2013, the US National Association of Emergency Medical Services Physicians took a position in favor of a judicious immobilization strategy [4].

Once on an ED stretcher, it is not unusual for these patients to remain fully immobilized for several hours until physician assessment and c-spine diagnostic imaging can be performed and interpreted. This consumes valuable time for physicians, nurses, and radiology technicians and distracts them from other urgent responsibilities. These delays compound the burden of our crowded Canadian EDs in an era when they are under unprecedented pressures. The median length of stay for a patient evaluated in the stretcher area is approximately 8 to 12 hours, whereas similar minor trauma victims arriving without immobilization can be evaluated and discharged in less than 4 hours.

#### Canadian C-Spine Rule

**Figure 1:**
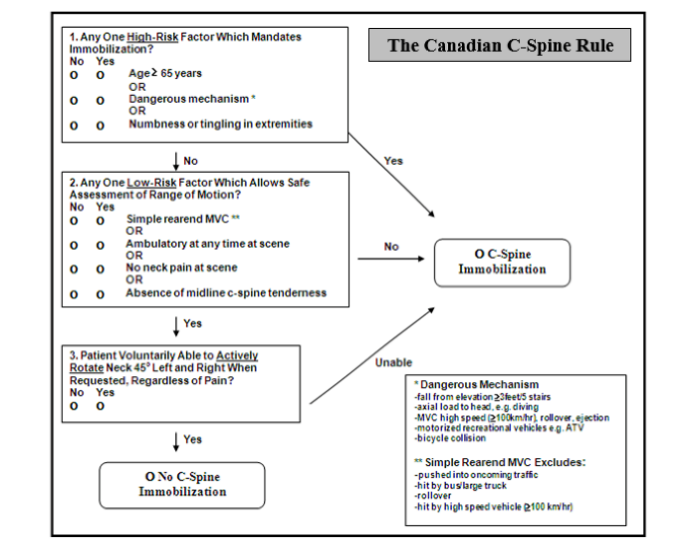
The Canadian C-Spine Rule adapted for use by paramedics.

We have derived, validated, and implemented the Canadian C-Spine Rule (CCR) to be used by physicians [5-7], triage nurses [8], and paramedics [9] in more than 40,000 alert and stable trauma patients. The CCR (Figure 1) directs that immobilization is unnecessary if the patient has no high-risk criteria, has at least one low-risk criterion, and can voluntarily rotate their neck 45° left and right. Physicians and nurses already use the CCR in the ED to safely remove immobilization devices without the need for imaging and with no documented adverse outcomes. We recently completed a pilot implementation study with Ottawa paramedics, where selective patients were transported without immobilization. We have recruited 3854 patients, and paramedics have identified all clinically significant injuries (100% sensitivity) without negative consequence when the CCR determined that immobilization was not required (68% specificity). Approximately 60% of immobilizations were avoided.

#### C-Spine Evaluation in Children

The National Emergency X-Radiography Utilization Study (NEXUS) decision instrument for use in adults and children was validated in 2160 children aged 8 to 17 years and identified all significant injuries [10]. The CCR’s performance was superior to that of the NEXUS decision instrument when prospectively compared in an adult population [6] but is yet to be implemented for use in children. A case-control study of children younger than 16 years with c-spine injuries identified 8 risk factors for significant injuries, 7 of which are included in the CCR [11].

On the basis of information provided by our paramedic stakeholders, we estimate that there are 4000 or more children aged 8 to 16 years transported with immobilization each year in Ontario. In a survey of physicians, 85% stated they would use the CCR if it were properly evaluated for use in the pediatric population [12].

#### Rationale for This Study

Minor trauma is very common, and these patients are usually transported to the ED by paramedics, but rarely do they have a fracture or spinal cord damage. Current immobilization and transport practice guidelines are not evidence-based, and there is a growing body of evidence testifying to the deleterious effects and consequences of this practice on patients, paramedic systems, EDs, and the health care system. We have successfully derived, validated, and implemented the CCR for use by physicians, nurses, and, more recently, by paramedics in a pilot project. Patient groups, paramedic stakeholders, ethics board members, and the Medical Advisory Committee for the Ontario Ministry of Health and long-term care Emergency Health Services Branch (EHSB) are all supportive of this multicenter implementation evaluation study. We now need a large pragmatic study to evaluate the feasibility, benefits, and safety of implementing the CCR in geographically and socially diverse prehospital communities. It is encouraging that most paramedic services would only participate in the study if we adopted a design that would guarantee, at some point, an opportunity for them to be assigned to the intervention arm and expand the scope of their paramedics’ practice.

### Objectives

The overall goals of this study are to improve patient care and health system efficiency and outcomes by allowing paramedics to assess eligible low-risk trauma patients with the CCR and selectively transport them *without* immobilization to the ED. We therefore sought to answer the following study question: Does allowing paramedics to assess selective low-risk trauma patients with the CCR and transporting them without immobilization result in *significant and immediate* health service benefits for patients, paramedic services, and EDs in a safe cost-effective manner?

## Methods

### Trial Design

The multicenter implementation of the CCR by paramedics is designed as a stepped-wedge cluster randomized trial with three sequences, involving a total of 13 Ontario paramedic services. Our 36-month study will consist of a 12-month setup and training period (year 1), followed by the stepped-wedge trial (year 2) and a 12-month period for study completion, analyses, and knowledge translation and exchange (see Table 1). Paramedic services in each sequence will cross from the control condition (usual care) to the intervention condition (CCR implementation) at intervals of 3 months until all communities have crossed to the intervention. Data will be collected on all eligible patients in each paramedic service for a total duration of 12 months.

**Table 1:** Diagram of the study and stepped-wedge design.

Year			Months 1-9	Months 10-12
Year 1			Study set-up ePlatform programming-paramedic data collection Preparation of study material and site visits	Paramedic training
**Year 2**				
	**Sequence**			
		1 (4 sites)	Months 1-3: Usual Care Months 4-6: CCR Months 7-9: CCR	CCR^a^
		2 (4 sites)	Months 1-3: Usual Care Months 4-6: Usual Care Months 7-9: CCR	CCR
		3 (4 sites)	Months 1-3: Usual Care Months 4-6: Usual Care Months 7-9: Usual Care	CCR
Year 3			Months 1-6: Study completion; data linkage with IC/ES^b^ Months 7-9: Data cleaning; data analyses	Reports and manuscripts writing; KTE^c^

^a^CCR: Canadian C-Spine Rule.

^b^IC/ES: Institute for Clinical Evaluative Sciences.

^c^KTE: Knowledge Translation and Exchange.

### Study Setting

The study will take place in the province of Ontario. Up to 12 new Ontario paramedic services will participate. Ottawa will also participate but only provide data for the pediatric cohort as the CCR has already been implemented within their practice. The 12 new paramedic services vary in terms of size, population served, and geographical location. Each paramedic service in Ontario is affiliated with a base hospital. There are eight regional base hospitals in Ontario that provide medical direction, leadership, and advice in the provision of prehospital emergency care. Although the base hospital programs will not be participating directly in the study as separate sites, they will be assisting with start-up, implementation, and follow-up.

### Population

All consecutive, alert (able to follow commands), stable patients (normal vital signs) will be evaluated by the paramedics employed by a participating paramedic service for potential cervical spine injury after sustaining acute blunt trauma (within 48 hours). These are patients for whom standard Ontario prehospital trauma protocols usually require immobilization. As in prior CCR studies, patients will be excluded if they do not require immobilization as per the standard Ontario paramedic trauma protocol, have a Glasgow Coma Scale score of less than 15 or are intubated, or have unstable vital signs (systolic blood pressure <90 mm Hg; respiratory rate <10 or >24 breaths/min). Patients will also be excluded if their injury occurred more than 48 hours earlier, if they have penetrating trauma from a stabbing or a gunshot wound to the neck, acute paralysis, or known vertebral disease (specifically ankylosing spondylitis, rheumatoid arthritis, spinal stenosis, or previous cervical spine surgery), if they were referred from another hospital and transported between facilities, or if they are younger than 8 years.

### Research Ethics Approval Process

The study protocol and all study-related documents (paramedic CCR and study data collection) have been approved by the Ottawa Health Sciences Network Research Ethics Board (OHSN-REB). The OHSN-REB has recently become a board of record for Clinical Trials Ontario. As a result, and because this is a multicenter study, the study protocol was submitted to the OHSN-REB through Clinical Trials Ontario. All participating sites that have existing agreements in place with Clinical Trials Ontario were included in the REB submission approval. We identified a local site investigator and helped coordinate REB submission, review, and approval for those sites that do not have agreements with Clinical Trials Ontario.

### Consent and Permissions

We obtained a waiver of patient-informed consent from the OHSN-REB, Clinical Trials Ontario, and all other participating research ethics boards. This was the case in the previous multicenter prehospital validation of the CCR and the single-center prehospital implementation study. The study protocol has been reviewed by the Medical Advisory Committee for the Ontario Base Hospitals Group (MAC-OBHG). The Medical Advisory Committee provides advice to the EHSB of the Ontario Ministry of Health and Long-Term Care. Paramedics employed by paramedic services participating in the study will be allowed via a medical directive to use the CCR to evaluate eligible patients instead of the usual immobilization protocols. The medical directive was drafted by the MAC-OBHG and authorized by the EHSB for the duration of the study.

### Paramedic Training

Paramedics will be trained in the use of the CCR before the start of the trial. We have conducted an Ottawa paramedic CCR implementation pilot study and have designed our training program to address barriers identified in the pilot. The training entails 1 hour of education: 30 min of self-review of a teaching video addressing the background and scientific development of the CCR and a 30-min in-class teaching video reviewing the specific steps involved in using the CCR, complete with a demonstration and question and answer period with a certified trainer. Paramedics will be *certified* to clear the cervical spine by medical directive if they have (1) successfully completed the initial training sessions and (2) successfully completed (score of ≥80%) a written quiz. Paramedics failing the written quiz would be required to attend a remedial session and review all wrong answers with their certified trainer. It should be noted that Ottawa paramedics all successfully completed their training.

During the study setup period (Table 1), each participating service will designate a local paramedic study champion. These individuals will be in close contact with staff at the study coordinating center and will receive further information about the study, methodology, and implementation of the CCR. These individuals will be heavily involved in delivering the study training material at their particular location and will serve as a first point of contact throughout the implementation. Paramedics with questions about specific aspects of the CCR or the application of the CCR for unusual scenarios will be able to communicate directly with a peer in an effort to promote adherence to the protocol. Paramedics will be encouraged to ask questions during the training sessions, speak directly with their study champion, add comments to study forms, or communicate with study staff via the study website or through social media. These questions and concerns will be compiled and distributed back to study champions to disseminate to local staff. Staff at the study coordinating center will regularly provide updates and reminders to study champions.

### Intervention

The stepped-wedge trial will begin after paramedic training has been completed (see Table 1). During the usual care phase, paramedics will complete the CCR data collection form for all eligible patients but will continue to immobilize them all before transport to the receiving hospital. Once a community has crossed to the intervention CCR phase, paramedics will be permitted by a medical directive to implement the CCR. Paramedics will then transport selected patients without immobilization according to the CCR. Although following the medical directive will be mandatory for paramedics, they will be encouraged and allowed to immobilize patients if they are uncomfortable with the CCR’s recommendation to not immobilize them.

### Outcome Measures

The outcomes of interest are divided into three categories: measures of patient and health system benefit, measures of patient benefit, and measures of health system benefit. These were supported and ranked by patients and paramedic stakeholders.

#### Measures of Patient and Health System Benefit

The measure of patient and health system benefit included the proportion of patients transported with immobilization (primary outcome).

#### Measures of Patient Benefit

The measures of patient benefit included the following:

1. Proportion of patients feeling comfortable (score ≤4 on a 10-point Likert scale; coprimary outcome)
2. Proportion of patients with a pain score ≤4 on a 10-point Likert scale upon transfer of care to the ED
3. Time from paramedic arrival to ED discharge or admission to hospital
4. Patient radiation exposure (in millisieverts) from diagnostic imaging of the spine
5. Number of skin pressure injuries
6. Number of missed clinically important c-spine injuries. A clinically important c-spine injury includes any injury other than the following defined unimportant injuries that require neither specialized treatment nor follow-up: isolated avulsion fracture of osteophyte, isolated fracture of the transverse process not involving the body or facet joint, isolated fracture of the spinous process not involving the lamina, isolated simple compression fracture less than 25% of body height.

#### Measures of Health System Benefit

The measures of health system benefit included the following:

1. Time spent in the field by paramedics before arrival to hospital
2. Time spent in the hospital by paramedics before transfer of care to the ED team
3. ED length of stay until discharge or admission to hospital
4. Number of subsequent ED visits or admission to hospital within 30 days of ED discharge
5. Number of subsequent clinic/family physician visits within 30 days of ED discharge
6. Frequency of c-spine diagnostic imaging performed within 30 days of ED discharge
7. Incremental cost per 1 immobilization avoided (including cost of training, equipment, paramedic time, ED utilization, diagnostic imaging, and follow-up visits)

### Data Collection and Data Sources

Once training of paramedic staff has been completed, paramedics will begin evaluating eligible patients with the CCR. Each time an eligible patient is assessed using the CCR, the paramedic treating that patient will complete and submit an electronic CCR. The paramedic will also record patient-reported comfort level and pain level on this form. Staff at the study coordinating center will receive the electronic paramedic-completed CCR and a copy of the electronic paramedic care record (ePCR). Study staff will review the paramedic documentation to assess compliance with the study protocol and application of the CCR. Information on patient age, gender, mechanism of injury, field time, offload time, and immobilization status are contained in the ePCR and will be recorded from there.

We will link the information obtained from paramedic care records to provincial administrative databases housed at the Institute for Clinical Evaluative Sciences (IC/ES). This linkage will allow us to obtain information related to the initial ED visit, c-spine diagnostic imaging, hospitalization, and subsequent ED or clinic or family physician visits within 30 days of injury.

### Confidentiality and Data Linkage

Paramedics will evaluate eligible patients using the CCR. They will complete an electronic form that will capture information on the elements of the CCR and pain, patient comfort, and paramedic comfort with using the CCR. The electronic form will not include any information that can identify a patient. Upon receipt of the electronic form, study staff will assign a unique study number. We will also receive the corresponding paramedic documentation electronically that will allow us to capture the remainder of the prehospital data required. The paramedic documentation will also be transmitted electronically, stripped of patient identifiers.

To link the prehospital information with the data housed at IC/ES, we will need to maintain a list of eligible enrolled patients, including first name, last name, date of birth, sex, postal code, and health card number (where available). This list will be generated and maintained by staff at each base hospital, or paramedic service if base hospital staff are unable to access this information. The information will be stored in a password-protected, encrypted spreadsheet. When this information is required by IC/ES for linkage purposes, it will be transmitted securely according to their protocols. The linked information that we receive back from IC/ES will be stripped of personal identifiers before we receive it. All paper study files will be stored in locked filing cabinets in a locked office. All electronic files will be stored on limited-access network folders that are backed up regularly. Any information shared with the study committees will not include any identifiable information.

### Data Management

Data will be entered centrally at the study coordinating center by trained study staff. Staff will receive training on the study protocol, definition of data elements, application of the CCR, and elements of the ePCR. A complete list of data points and definitions will be compiled and included in a study manual for reference. The data will be entered electronically. The data entry screens will resemble the paper study forms approved by the steering committee. Where possible, the study database will be designed to ensure that each given variable can only be entered in a certain format, thereby limiting the number of errors in data entry. A certain percentage of cases will be entered in duplicate to ensure accuracy. A small percentage of cases (10%) will also be pulled and compared with the source documents to independently verify the accuracy of the data. We will regularly run range and logic checks to previously entered data to locate and fix any errors or discrepancies in the data set. We will work closely with the staff at the participating base hospitals and our local paramedic study champions to promptly identify and locate missing data. Queries about particular cases and situations will be flagged for review by the research coordinator. If the research coordinator is unable to determine the appropriate course of action, the flagged issue will be brought to the attention of the principal investigator who will review the issue and advise. Any resulting changes to data definitions will be noted and dated in the study manual.

The study database will be designed and located on servers housed at the Ottawa Hospital Research Institute. All electronic study documents will be saved on network folders with limited access. The network folders are backed up nightly by the Ottawa Hospital Research Network Information Technology team. Paper files will be stored in locked cabinets in locked offices.

### Auditing

We plan to conduct regular site visits with all participating sites. The initial visit will be primarily to go over training material with local study staff, go over study requirements, and ensure local study staff have all the necessary study documentation. The intervention duration is 12 months. We will conduct one subsequent visit to each site during the invention phase to ensure that study documentation is accurate and up to date, all study material is accurate and up to date, and local study procedures are being conducted as per the study protocol. If concerns are noted, we will work individually with each site to address the concern and rectify the situation.

### Sample Size

Our sample size for this study is determined mainly by pragmatic considerations: we need a large number of sites from across Ontario to evaluate the safety and generalizability of the implementation in this multicenter setting while accounting for between-site differences such as size and setting. Power calculations were carried out for the stepped-wedge trial. Using data from a previous study in these communities, we expect approximately 600 patients per paramedic service per year (or 150 patients per 3-month time interval). A total of 12 paramedic services (7200 patients in total) evaluated across four time intervals in a stepped-wedge design will provide adequate power to detect minimally important differences of 10% in our two coprimary outcomes using two-sided tests at the 2.5% level of significance. In particular, for our *primary outcome,* we will have greater than 99.9% power to detect a minimally important absolute reduction of 10% in the proportion of patients immobilized, assuming a control arm proportion of 1. For our *coprimary outcome,* we will have 80% power to detect a minimally important increase of 10% in the proportion of patients feeling comfortable assuming a conservative control arm proportion of 0.5. In these calculations, we have assumed a commonly used within-period intracluster correlation coefficient of 0.05, and an exponential decay with a decay parameter of 0.85 (ie, a 15% decay per period).

### Recruitment Feasibility

On the basis of the volume of immobilized patients transported in each of the 12 new proposed participating centers, we expect there could be 8129 eligible cases over the proposed 12-month evaluative period (required sample size is 7200). We are confident that the required sample size can be obtained with the participation of the proposed centers, and we have accounted for unlikely attrition in our study design and sample size calculation.

We also plan to employ a number of strategies during the enrollment phase of the study to meet our recruitment goals. We have specifically approached Ontario paramedic services that have previously and successfully participated in prehospital research. These paramedics will be familiar with completing specific study paperwork. We will be approaching the vendors of the software used by paramedic services to develop a study form that is easy to access, complete, and submit. We will employ a local study champion at each paramedic service who will be accessible to the frontline paramedics to answer questions, deliver updates and reminders, and provide feedback regarding certain cases or applications of the CCR. Finally, we will develop a study website and utilize social media to keep the participating paramedic services and their staff engaged in the study.

### Randomization and Allocation

The 12 new participating paramedic services will be randomized using the technique of covariate constrained allocation to protect against chance imbalances in the following prognostic factors: catchment area (km^2^), number of immobilizations per month, average response time, and staff makeup (advanced care paramedics and primary care paramedics). Owing to the relatively small number of allocation units, it is particularly important to use an allocation technique that minimizes the risk of chance imbalances. In the stepped-wedge design, randomization is with respect to the timing of implementation of the intervention. Effective randomization is essential to protect the internal validity of the trial, including the ability to obtain a valid estimate of any secular trend and a valid estimate of the intervention effect. Covariate constrained allocation was selected as it was found to be superior to simple stratification and matching in a recent simulation study [13]. In covariate constrained allocation, all possible allocations of sites will be considered (a total of 34,650 possible allocations) and those that are acceptable, in that they meet a set of balance constraints, will be identified. One of the allocations will then be randomly selected from among the set of acceptable allocations. To protect the validity of the randomization, the number of times that any given pair of sites receives the same allocation will be counted, and constraints will be relaxed if the design is found to be overly constrained. The allocation will be performed using a SAS macro developed for this purpose, by an independent statistician not associated with the trial [14]. Allocations will be securely kept by the independent statistician and will be concealed from the study investigators and all participating sites until 1 month before the allocated start time of a particular site.

### Statistical Analyses

Analyses will be conducted at the level of the individual patient using generalized linear mixed-effects regression, with random effects to account for clustering by paramedic service and over time and fixed effects for treatment and time interval to account for the stepped-wedge design. The analysis will adjust for sex, age, and the need for immobilization according to the CCR. The primary and coprimary outcomes will be analyzed using binomial distribution with identity, log, or logit link, and the effect of intervention will be expressed as absolute differences, relative risk, or odds ratios with 97.5% CIs. Secondary outcomes will be similarly analyzed using binomial distribution and identity or logit link for dichotomous variables, normal distribution and identity link after log transformation or gamma distribution and log link for continuous variables with a skewed distribution, or Poisson or negative binomial distribution with log link for count variables. The effect of the intervention on each secondary outcome will be described using absolute difference, relative risk, or odds ratio with 95% CI. Subgroup analyses (described in the following sections) will be conducted by including interactions with time interval and treatment in the regression model.

Our *health economist* will perform a cost-effectiveness analysis from the perspective of the Ministry of Health and Long-Term Care. Trial data will be used to populate the relative costs and outcomes of the use of the CCR by paramedics with usual care (100% immobilization). Resource use will be collected during the trial and obtained from IC/ES, whereas unit costs will be obtained from appropriate Canadian sources, such as Schedule of Benefits for Physician Services. The total cost for each patient includes the costs of the intervention and costs of health services, including the follow-up period of 30 days post-ED discharge. The cost of intervention covers the cost of training and operation. Costs of operating paramedic services include personnel cost (eg, salaries and employee benefits), service cost (eg, fuel and maintenance), medical supplies (eg, an onboard liquid oxygen system, medications, and single-use patient care supplies). The costs of health care services will be obtained from IC/ES and will be estimated by multiplying the unit costs by the volume of health care used. We will use mixed-effects regression analyses to estimate the difference in expected health care costs and outcomes between the intervention and control groups. The incremental cost-effectiveness ratio will be estimated by dividing a difference in cost by a difference in the number of immobilizations. The 95% CI will be calculated using a nonparametric bootstrapping method. Results from the bootstrapping exercise will also be used to depict a cost-effectiveness acceptability curve (CEAC), which links the probability of a treatment being cost-effective to a range of potential threshold values (lambda) that the health system may be willing to pay for an additional unit of effect [15]. A CEAC is a graphical representation of the probability that the CCR may be cost-effective given the alternate dollar values placed on an outcome. This will allow estimation of the probability that the CCR can be considered cost-effective given the available data. In addition, sensitivity analysis will be undertaken to examine the effect of conducting a complete case-only analysis and of varying the cost of the intervention. We will also conduct a budget impact analysis to estimate the financial consequences of implementing the CCR by paramedics in Ontario*.* All analyses will be conducted using STATA version 13.0 and Microsoft Excel and Visual Basic for Applications.

*Prespecified subgroup analyses* will be conducted to examine the differential effects (possible inequity) of the intervention on the following groups, defined by the following:

1. Sex
2. Language barrier present vs not present (collected by paramedics on data collection form)
3. Long transport times (longer vs shorter than 15 min)
4. Age (adult ≥16 years vs children <16 years)
5. Socioeconomic status and education level (IC/ES data)
6. Type of backboard used (full board, open-back scoop, or trunk and neck Kendrick extrication device)

## Results

We received study funding in 2015 and institutional research ethics approval in 2016. Recruitment and data collection took place between March 2017 and May 2018 and included a total of 6049 patients at the time of submission. Data linkage and analyses are under way, and the final results are expected in the spring of 2020. If this multicenter trial is successful, we expect that the Ontario Ministry of Health will recommend that paramedics evaluate all eligible patients with the CCR in the Province of Ontario.

## Discussion

We conservatively estimate that in Ontario, more than 60% of all eligible trauma patients (300,000 annually) could be transported safely and comfortably, without c-spine immobilization devices. This will significantly reduce patient pain and discomfort, paramedic intervention times, and ED length of stay, thereby improving access to paramedic and ED care. This could be achieved rapidly and with lower health care costs compared with current practices (possible cost saving of Can $36 [US $25] per immobilization or Can $10,656,000 [US $7,335,231] per year).

In addition, this project will facilitate a new paradigm in prehospital research by integrating paramedics and patients actively into the research and knowledge translation process. It will offer the added benefits of consolidating a network of paramedic research partners and of facilitating future collaborative projects. It will also make innovative use of data provided by the IC/ES to streamline and decrease the cost of conducting prehospital research, and, with the help of our new partners, foster collaborative efforts to measure and possibly correct health inequities in prehospital care. Finally, this project could lead to the use of the CCR by paramedics from across Ontario and Canada and to immediate health care benefits/savings on a national scale.

## Appendix

Multimedia Appendix 1 Peer-review and Responses to reviewers.

## Abbreviations

CCR: Canadian C-Spine Rule
CEAC: cost-effectiveness acceptability curve
ED: emergency department
EHSB: Emergency Health Services Branch
ePCR: electronic paramedic care record
IC/ES: Institute for Clinical Evaluative Sciences
ILCOR: International Liaison Committee on Resuscitation
KTE: Knowledge Translation and Exchange
MAC-OBHG: Medical Advisory Committee for the Ontario Base Hospitals Group
NEXUS: National Emergency X-Radiography Utilization Study
OHSN-REB: Ottawa Health Sciences Network Research Ethics Board
