# Supplementary material for: Pragmatic Strategy Empowering Paramedics to Assess Low-Risk Trauma Patients With the Canadian C-Spine Rule and Selectively Transport Them Without Immobilization: Protocol for a Stepped-Wedge Cluster Randomized Trial
Source: JMIR Res Protoc. 2020 Jun 1;9(6):e16966. doi: 10.2196/16966 (PMC7296410; doi:10.2196/16966)
Supplement: Multimedia Appendix 1 [file resprot_v9i6e16966_app1.pdf]

From: Mark Roseman <markroseman@ossu.ca>
Sent: March 2, 2015 11:56 AM
To: Vaillancourt, Christian <CVaillancourt@ohri.ca>
Cc: Carolyn Genautis <CarolynGenautis@ossu.ca>
Subject: OSSU IMPACT Awards Feedback and Full Application Form

Dear Christian,

Following on Vasanthi's email last week, let me congratulate you on the selection of your Letter of Intent (LOI) to proceed to the full application phase of the IMPACT Awards competition.

Please note that the full application must be submitted via e-mail to ossu@ossu.ca no later than **5:00pm (EST) on Monday April 27, 2015.**

To assist you in the full application phase, you have been invited to attend a one-day **Strengthening Workshop** on **Friday, March 13, 2015** at the Chestnut Residence and Conference Centre, 89 Chestnut Street, Toronto. If you have not already done so, please RSVP to Carolyn Genautis at carolyngenautis@ossu.ca as soon as possible to confirm your attendance.

As well, the following feedback from the LOI Review Panel is provided to help inform the development of your full application:

- The reviewers felt that this was an important study proposed by a very strong and competent team. There is already convincing evidence that the premise of the proposal works based on the evaluation of more than 3,000 Ottawa-based patients. The current proposal will extend that study to several additional Ontario centres, potentially leading to improved patient care and a revision of medical practice guidelines. To strengthen the full application, the reviewers had the following suggestions:

    - In the full application, reviewers would like to see a convincing argument as to why this study could not be accomplished as effectively through a targeted training program based on the Ottawa evidence.
    - It will also be important to demonstrate real cost savings in emergency room usage.
    - Any overlap with other grants will need to be fully described and clarified.
    - Patient engagement will need to be strengthened.

Congratulations once again, and I look forward to seeing you at the workshop on March 13.

Best regards,

Mark

Mark Roseman, Ph.D., MBA
Director, Ontario SPOR SUPPORT Unit (OSSU)

MaRS Centre, West Tower
661 University Ave., Suite 405
Toronto, ON M5G 1M1

Tel: 416-673-8431
Email: markroseman@ossu.ca

Responses to Review Panel Feedback

"The reviewers felt that this was an important study proposed by a very strong and competent team. There is already convincing evidence that the premise of the proposal works based on the evaluation of more than 3,000 Ottawa-based patients. The current proposal will extend that study to several additional Ontario centres, potentially leading to improved patient care and a revision of medical practice guidelines."

>Applicants: We thank the reviewers for these very generous comments.

"To strengthen the full application, the reviewers had the following suggestions:"

- *"In the full application, reviewers would like to see a convincing argument as to why this study could not be accomplished as effectively through a targeted training program based on the Ottawa evidence."*

  > Indeed, the experience we have had with our pilot implementation in Ottawa was successful. However, this only represents the experience of a single urban center whose paramedics have a long-standing culture of participation in research protocols. From our past large-scale research experiences (In-hospital use of the CCR by physicians and nurses, and the Ottawa Ankle Rule), multi-community studies are essential to ensure proper evaluation and adoption. Our stepped-wedge design also ensures successful uptake of our strategy in each of the communities, provided we indeed demonstrate its effectiveness. A multi-centre design is required to evaluate safety and generalizability of implementation while accounting for between-site differences in size, and urban and rural settings.

  We also met with the MOHLTC-EHS's Medical Advisory Committee of physicians responsible for recommendations pertaining to paramedicine practice in Ontario. Their opinion on the use of the CCR was quote: *"We believe further implementation data is required before we can recommend making changes to the current provincial medical directive."* We were told the current proposal, if successful, would satisfy their requirement.

Furthermore, Dr. Prpic, Chair of the MOHLTC-EHS's Medical Advisory Committee goes on to say: *"Should Dr. Vaillancourt not be able to secure funding and expand this important project to additional study sites, the MAC will be faced with the very difficult task of having to make recommendations based solely on preliminary and inconclusive data from the pilot project in Ottawa. This is not a position that we are comfortable with, and the patients of Ontario would benefit from a definitive answer to the question of prehospital C-spine clearance."*

- *"It will also be important to demonstrate real cost savings in emergency room usage."*

  >Our proposed analyses on health care savings include the costs involved during the prehospital care of patients, the cost/impact on their ED utilization, in addition to potential health care utilization costs following their ED discharge (such as subsequent diagnostic imaging or clinic visits).

- *"Any overlap with other grants will need to be fully described and clarified."*

  >We originally had the intention to submit an application to the CIHR transitional award competition, but, following our invitation to submit a full application to the OSSU IMPACT Award competition, we have decided to put all our efforts in the preparation of this proposal. There will be no overlap with any other grant (present or future).

- *"Patient engagement will need to be strengthened."*

  >We have made significant efforts to collaborate with OSSU's experts on patient engagement, have implemented the OSSU framework on patient engagement, have identified and welcomed a patient representative on our study team, and are recruiting additional representatives to sit on our Patient Committee. Details of our patient engagement plan are presented in Section 7, and incorporated throughout most sections of this proposal.
